# Integration of GPS Precise Point Positioning and MEMS-Based INS Using Unscented Particle Filter

**DOI:** 10.3390/s150407228

**Published:** 2015-03-25

**Authors:** Mahmoud Abd Rabbou, Ahmed El-Rabbany

**Affiliations:** Department of Civil Engineering, Ryerson University, 350 Victoria Street, Toronto M5B 2K3, ON, Canada; E-Mail: rabbany@ryerson.ca

**Keywords:** GPS, PPP, INS, EKF, UKF, UPF, tightly coupled

## Abstract

Integration of Global Positioning System (GPS) and Inertial Navigation System (INS) integrated system involves nonlinear motion state and measurement models. However, the extended Kalman filter (EKF) is commonly used as the estimation filter, which might lead to solution divergence. This is usually encountered during GPS outages, when low-cost micro-electro-mechanical sensors (MEMS) inertial sensors are used. To enhance the navigation system performance, alternatives to the standard EKF should be considered. Particle filtering (PF) is commonly considered as a nonlinear estimation technique to accommodate severe MEMS inertial sensor biases and noise behavior. However, the computation burden of PF limits its use. In this study, an improved version of PF, the unscented particle filter (UPF), is utilized, which combines the unscented Kalman filter (UKF) and PF for the integration of GPS precise point positioning and MEMS-based inertial systems. The proposed filter is examined and compared with traditional estimation filters, namely EKF, UKF and PF. Tightly coupled mechanization is adopted, which is developed in the raw GPS and INS measurement domain. Un-differenced ionosphere-free linear combinations of pseudorange and carrier-phase measurements are used for PPP. The performance of the UPF is analyzed using a real test scenario in downtown Kingston, Ontario. It is shown that the use of UPF reduces the number of samples needed to produce an accurate solution, in comparison with the traditional PF, which in turn reduces the processing time. In addition, UPF enhances the positioning accuracy by up to 15% during GPS outages, in comparison with EKF. However, all filters produce comparable results when the GPS measurement updates are available.

## 1. Introduction

Traditionally, differential GPS with tactical or navigation-grade inertial sensors are used in Global Positioning System (GPS) and Inertial Navigation System (INS) integration for precise navigation applications [1,2,3,4]. This is mainly due to the inherited high accuracy of differential GPS in comparison with the standalone GPS mode. Unfortunately, this requires a relatively nearby base station, which limits the navigation range and increases the cost and complexity of the system. The precise point positioning (PPP) technique is capable of providing decimeter-positioning accuracy without the need for a base receiver [5]. PPP has been the focus of a number of research groups in the last two decades (see for example, [6,7,8]). To speed up the PPP solution convergence time, a number of PPP ambiguity resolution techniques have been developed [9,10,11]. PPP has been used in a number of applications, including precise surveying, disaster monitoring, offshore exploration, and others [12,13,14]. On the inertial side, recent advances in micro-electro-mechanical sensors (MEMS) technology enabled the development of a generation of low-cost inertial sensors. MEMS sensors are characterized by their small size, light weight and low cost, in comparison with high-end inertial sensors. However, MEMS sensors generally have poorer performance compared with high-end inertial navigation unit (IMU) due to the significantly higher errors and biases affecting these low-cost inertial sensors.

Commonly, the extended Kalman filter (EKF) is considered as the estimation filter for GPS/INS integration (e.g., [3,4,15]). In EKF, the non-linear system and observation models are linearized around the updated navigation parameters using the first-order Taylor series expansion, under the assumption that the noise is Gaussian. However, as a result of neglecting higher order terms, EKF might fail to produce a reliable estimation solution, especially during GPS outages. This is particularly the case when low-cost MEMS-based inertial measurement units (IMU) are used. The iterated extended Kalman filter (IEKF) was considered by a number of researchers, e.g., [16,17] which attempts to improve the linear approximation of the observation model through iterative re-linearization. Unfortunately, the IEKF does not overcome the convergence problem completely.

The unscented Kalman filter (UKF) was introduced by [18] as a linear regression estimation filter. UKF propagates a deterministically a fixed set of sigma points with appropriate weights through the non-linear motion and observation models to capture the system a posteriori mean and covariance estimates [19]. However, similar to EKF, the algorithm is still dealing with the assumption of Gaussian distribution. In contrast to linearization filters, Particle filtering (PF) avoids the linearization of the system models. Rather, it obtains an approximate estimation solution for the nonlinear model. In addition, PF can accommodate non-Gaussian distributions noise. As a result, it can be considered as a non-parametric estimation method for non-linear/non-Gaussian applications. A drawback of the PF, however, is that it is featured by a large computational cost, which represents the main limitation in practical use. Nevertheless, with the advances in computer technology, a number of researchers successfully used it for GPS/INS integration (e.g., [20,21,22,23]).

To overcome the linearization and computational cost problems, recent research focused on fusing PF with either of EKF or UKF to form the extended particle filter (EPF) or the unscented particle filter (UPF), respectively [24,25]. Haug [24] used EKF or UKF to produce a posteriori mean and covariance estimates, which are then employed to produce the PF importance density function for particle generation. Then, the normalized importance weights of the particles are calculated to refine the system *posteriori* estimates. Although, this technique significantly reduces the number of particles and processing time compared with traditional PF, it confines the PF importance density function to Gaussian distribution. As such, the expected enhancement can be considered limited [26]. According to Simon [25], a bank of EKFs or UKFs can used for each particle combined with the likelihood function to derive the system *a posteriori* estimates. This technique can significantly reduce the number of needed particles while reserving the non-Gaussian natural of the system noise.

In this research, a UPF is developed, based on the approach proposed by Simon [25], to merge GPS measurements, through un-differenced PPP technique, and the inertial sensor measurements. All of the available GPS observations, including pseudorange, carrier-phase, and corrected Doppler observations, are used. The performance of the developed filter is compared with that of the traditional filters, including the standard EKF, UKF, and PF, both when GPS is available and when there is a complete GPS outage, are encountered. It is shown that, as long as no GPS outages are encountered, the performance of all estimation filters is comparable. However, during GPS outages, the performance of UPF is superior to the traditional estimation filters. On average, about 15% positioning accuracy enhancement is obtained through UPF, in comparison with EKF. In addition, the number of particles needed to capture an accurate estimation is significantly reduced when UPF is used, in comparison with the traditional PF, which in turn reduces the computational cost significantly.

## 2. GPS PPP/MEMS Measurement and Motion Models

Tightly coupled (TC) architecture is implemented in this research, adopting a central filter to process the GPS raw measurements (pseudorange, carrier-phase, and Doppler) and the IMU measurements to produce estimates of the state vector including position, velocity, and attitude. The mathematical model of the inertial navigation system is commonly described in the framework of linear dynamic systems. The dynamic behavior of such systems can be described using a state-space representation. For this purpose, a system of non-linear first-order differential equations can be described as [27]:

(1) $$\left[\begin{array}{c} {\dot{r}}^{n}\\ {\dot{V}}^{n}\\ {{\dot{R}}_{b}}^{n} \end{array}\right]=\left[\begin{array}{c} D\cdot V^{n}\\ {R_{b}}^{n}f^{b}-(2{\Omega^{n}}_{ie}+{\Omega^{n}}_{en})\cdot V^{n}+g^{n}\\ {R_{b}}^{n}({\Omega^{n}}_{ib}-{\Omega^{b}}_{in}) \end{array}\right]$$

where, ${\text{r}}^{\text{n}}$ is the position vector, latitude, longitude and altitude; ${\text{V}}^{\text{n}}$ is the velocity vector in the East, North and Up (ENU) reference frame, ${\dot{\text{V}}}^{\text{n}}$ is the kinematic acceleration vector in the ENU reference frame; ${\text{Ω}}_{\text{en}}^{\text{n}}.\;{\text{V}}^{\text{n}}$ represents the effect of the motion of the ENU frame with respect to the ECEF frame; $2{\text{Ω}}_{\text{ie}}^{\text{n}}.\;{\text{V}}^{\text{n}}$ is the Coriolis acceleration vector; ${\text{g}}^{\text{n}}$ is the gravity vector, including the gravitation term and the centripetal term related to the Earth rotation; and ${\text{f}}^{\text{b}}$ is the specific force vector in the body frame, which is measured by the accelerometers. The matrix ${\text{Ω}}_{\text{ie}}^{\text{n}}$ is the skew-symmetric matrix of rotation rate vector of the Earth, which can be expressed in the ENU frame as:

(2) $$\Omega_{ie}^{n}=\left[\begin{array}{ccc} 0 & 0 & 0\\ 0 & \omega cos\varphi & 0\\ 0 & 0 & \omega sin\varphi \end{array}\right]$$

The matrix ${\text{Ω}}_{\text{en}}^{\text{n}}$ is the skew-symmetric matrix of the rotation rate vector of the ENU frame with respect to ECEF frame, expressed in the ENU frame as:

(3) $$\Omega_{en}^{n}=\left[\begin{array}{ccc} \frac{-V_{n}}{M+h} & 0 & 0\\ 0 & \frac{V_{E}}{N+h} & 0\\ 0 & 0 & \frac{V_{E}tan\varphi }{N+h} \end{array}\right]$$

The matrix ${\text{Ω}}_{\text{ib}}^{\text{b}}$ is the skew-symmetric matrix of the rotation rate vector of the body frame with respect to the ECI frame ${\text{ω}}_{\text{ib}}^{\text{b}}$, expressed in the body reference, which is measured by the gyros. The matrix ${\text{Ω}}_{\text{in}}^{\text{b}}$ is the skew-symmetric matrix of the rotation rate of the navigation frame with respect to inertial frame ${\text{ω}}_{\text{in}}^{\text{b}}$ expressed in the body frame, which is computed combining ${\text{ω}}_{\text{ie}}^{\text{n}}$ and ${\text{ω}}_{\text{en}}^{\text{n}}$ transforming the result in the body frame as follows:

(4) $${\Omega_{in}}^{b}={R_{n}}^{b}\cdot ({\Omega_{ie}}^{n}+{\Omega_{en}}^{n})$$

The bias and scale factor drifts are modeled as a first-order Gauss-Markov process, which can be formed as follows:

(5) $$\delta {\dot{b}}_{ai}=-\frac{1}{\tau_{bai}}\delta b_{ai}+w_{bai}$$

(6) $$\delta {\dot{b}}_{gi}=-\frac{1}{\tau_{bgi}}\delta b_{gi}+w_{bgi}$$

(7) $$\delta {\dot{S}}_{ai}=-\frac{1}{\tau_{Sai}}\delta S_{ai}+w_{Sai}$$

(8) $$\delta {\dot{S}}_{gi}=-\frac{1}{\tau_{Sgi}}\delta S_{gi}+w_{Sgi}$$

where the subscript *“i*” indicates the axis; $\tau_{a}$ and $\tau_{g}$ are the correlation times for the accelerometers and gyros, respectively; and $w_{a}$ and $w_{g}$ are the Gauss-Markov process driving noises, of which spectral densities are $q_{a}$ and $q_{g}$. The clock errors unique to the GPS measurements, including the clock offset and drift are modeled by [28]:

(9) $$\delta (c\dot{\delta }t_{offset})=\delta (c\delta t_{drift})+w_{offset}$$

(10) $$\delta (c\dot{\delta }t_{drift})=w_{drift}$$

where $w_{offset}$ and $w_{drift}$ are the clock offset and drift driving noise with spectral density $q_{offset}$ and $q_{drift}$, respectively. The measurement model of the GPS/INS filter in the TC architecture has the typical form:

(11) $$y_{k}=h(x_{k})+w$$

where $y_{k}$ is the corrected un-differenced ionosphere-free GPS measurements; *h(x_k_)* is the nonlinear measurement model which relates the stated vector x with the observation vector y and *w* is the Gaussian white noise with zero mean and covariance matrix ${\text{P}}_{\text{y}}$.

The mathematical model for the un-differenced ionosphere-free combination of code and carrier phase measurements can be written as:

(12) $${\text{P}}_{3}\text{=}\frac{{f_{1}}^{2}P_{1}-{f_{2}}^{2}P_{2}}{{f_{1}}^{2}-{f_{2}}^{2}}=\rho \;+cdt_{r}{-cdt}^{s}{+T+c(Ad}_{r\text{1}}{-Bd}_{r2})-{c(Ad}^{s1}{-Bd}^{s2}\text{)+}e$$

(13) $$\Phi_{3}\text{=}\frac{{f_{1}}^{2}\Phi_{1}{{-f}_{2}}^{2}\Phi_{2}}{{f_{1}}^{2}{{-f}_{2}}^{2}}{=\rho \;+cdt}_{r}{-cdt}^{s}{+T+c+(A\delta }_{r1}{-B\delta }_{r2})-{c(A\delta }^{s1}{-B\delta }^{s2})+(\bar{\lambda }\bar{N})+\varepsilon$$

where $P_{1}$ and $P_{2}$ are GNSS pseudorange measurements on $L_{1}$ and $L_{2}$, respectively; $\Phi_{1}$ and $\Phi_{2}$ are the GNSS carrier phase measurements on $L_{1}$ and $L_{2}$, respectively; $dt_{r}$ and $dt^{s}$ are the clock errors for receiver and satellite, respectively; $d_{r}$ and $d^{\text{s}}$ are frequency-dependent code hardware delay for receiver and satellite, respectively; $\delta_{r}$ and $\delta^{s}$ are frequency-dependent carrier phase hardware delay for receiver and satellite, respectively; *e*, *ε* are relevant system noise and un-modeled residual errors; and $\bar{\lambda N}$ is the ambiguity term for phase measurements. For the un-differenced ionosphere free linear combination, this term is not integer due to the non-integer nature of the combination coefficients, $\bar{\lambda N}=\frac{f_{1}^{2}\lambda_{1}N_{1}-f_{2}^{2}\lambda_{2}N_{2}}{f_{1}^{2}-f_{2}^{2}}$, *w*here $N_{1}$ and $N_{2}$ are the $L_{1}$ and $L_{2}$ non-integer ambiguity parameters, including the initial phase biases at the satellite and the receiver, respectively; $\lambda_{1}$ and $\lambda_{2}$ are the wavelengths of the ${\text{L}}_{1}$ and ${\text{L}}_{2}$ carrier frequencies, respectively; c is the speed of light in vacuum; *T* is the tropospheric delay component; ρ is the true geometric range from the antenna phase center of the receiver at reception time to the antenna phase center of the satellite at transmission time. *A* and *B* are frequency dependent factors $A=\frac{f_{1}^{2}}{f_{1}^{2}-f_{2}^{2}}$ and $B=\frac{f_{2}^{2}}{f_{1}^{2}-f_{2}^{2}}$.

**Figure 1:**
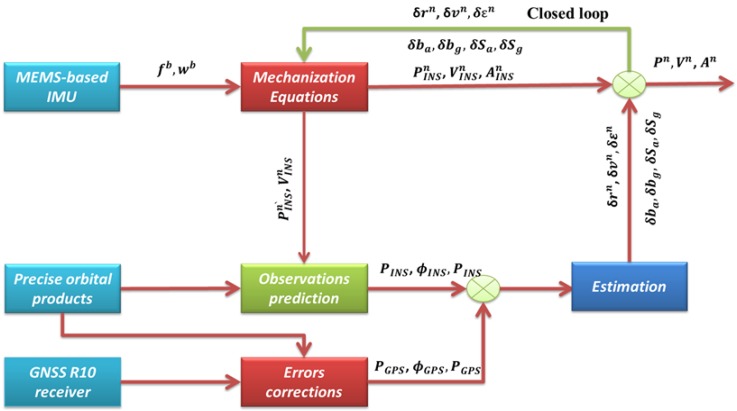
Flow chart of the proposed tightly coupled GPS PPP/MEMS integrated system.

With the availability of the final IGS orbital products corrected for the effect of the Earth’s rotation during signal transit, the outputs of position and velocity from the INS mechanization are used to predict the pseudorange, phase, and Doppler measurements through the non-linear observation equations. The UNB3 tropospheric model, consisting of the Saastamoinen vertical propagation delay model and Niell mapping function, is used to account for the tropospheric error [29]. The effects of ocean loading, Earth tide, carrier-phase windup, sagnac, relativity, and satellite antenna phase-center variations are accounted for using existing models [30]. In addition, the satellite clock error is accounted for using the final IGS clock products. Considering the above corrections, the corrected pseudorange, carrier phase and Doppler measurements from GPS, as well as the INS-predicted measurements, are processed by the integration filter to estimate the INS state vector. Finally, the obtained INS state estimates are fed back to the INS mechanization using the closed loop approach. The architecture of the proposed tightly coupled integrated system is shown in Figure 1.

## 3. Estimation Filters

Nonlinear estimation filtering techniques are employed to estimate the state vector of the proposed integrated GPS PPP/MEMS-based inertial system. In this section, the algorithms of UKF and PF are first briefly described. Then, the proposed unscented particle filter (UPF) is introduced.

### 3.1. Unscented Kalman Filter (UKF)

In UKF, number of points with appropriate weights called sigma points are deterministically selected to simulate the system probability density function under the assumption of Gaussian distribution. According to Bergman [19], the sigma points can capture the mean and covariance of a random vector up to the third order accuracy. Comparing with the traditional EKF, in which the higher order terms in Tylor expansion series are neglected, UKF should provide superior performance in simulating the Gaussian distribution and the nonlinearity behavior of the systems. The sigma points with zero mean can be generated based on a given squared dimension covariance matrix. As our distribution has a desired mean $\bar{x}$, a symmetric of 2n points is generated around the mean state vector. The generated points are propagated through the motion model yielding the predicted mean and covariance. Finally, the updated mean and covariance are estimated based on the GPS observations updating. The unscented Kalman filter can be defined according to [19] follows:
*1*.*Initialize with (k=0);*$${\bar{x}}_{0}=E[x_{0}]$$
$P_{0}=E[(x_{0}-{\bar{x}}_{0}){\left(x_{0}-{\bar{x}}_{0}\right)}^{T}]$*2*.*Define Sigma Points*;
$$x_{k-1}^{i}=\bar{x}+\sqrt{(n+\lambda )P_{x}},W_{i}=\frac{1}{2(n+\lambda )}$$
$$x_{k-1}^{i+n}=\bar{x}-\sqrt{(n+\lambda )P_{x}},W_{i+n}=\frac{1}{2(n+\lambda )}$$
Where *I = 1: n*, are the sigma points and *n* is the dimension of the state vector. The parameter λ is a scaling parameter.*3*.*Motion Model Update Step*;$x_{k,k-1}^{i}=f(x_{k-1}^{i},u_{i})+w_{k}$*4*.*Measurement Update*;
$$Z_{k,k-1}^{i}=h(x_{k,k-1}^{i}),{\bar{Z}}_{k,k-1}=\sum_{i=0}^{2n}W_{i}Z_{k,k-1}^{i}$$
$${\bar{x}}_{k,k-1}=\sum_{i=0}^{2n}W_{i}x_{k,k-1}^{i}$$
$$P_{z_{k}}=\sum_{i=0}^{2n}W_{i}({\bar{Z}}_{k,k-1}-Z_{k,k-1}^{i}){\left({\bar{Z}}_{k,k-1}-Z_{k,k-1}^{i}\right)}^{T}$$
$$P_{x_{k,k-1}}=\sum_{i=0}^{2n}W_{i}({\bar{x}}_{k,k-1}-x_{k,k-1}^{i}){\left({\bar{x}}_{k,k-1}-x_{k,k-1}^{i}\right)}^{T}$$
$$K_{k}=P_{x_{k,k-1}}{P_{y_{k}}}^{T}$$
$${\bar{x}}_{k}={\bar{x}}_{k,k-1}+K_{k}{\bar{Z}}_{k,k-1}$$
$$P_{x_{k}}=P_{x_{k,k-1}}-K_{k}P_{z_{k}}{K_{k}}^{T}$$
where ${\bar{x}}_{0}$ and $P_{0}$ are the initial state vector and variance-covariance matrix, respectively; $x^{i}$ and $Z^{i}$ are the state and observation vectors for the corresponding sigma points; *f* and *h* are the non-linear motion and observation models, respectively; $x_{k,k-1}$, $Z_{k,k-1}$ and $P_{x_{k,k-1}}$ are the time prediction state vector, observation vector and variance-covariance matrix, respectively; $x_{k}$, and $P_{x_{k}}$ are the time update state vector and variance-covariance matrix, respectively.

### 3.2. Particle Filtering (PF)

In contrast with the deterministic sigma points which are simulating Gaussian probability assumption in UKF, PF uses Monte Carlo simulation technique to approximate the non-Gaussian probability distribution through a set of weighted samples called particles around the mean state vector $\bar{x}$ [31]. The simulated particles are propagated through the non-linear motion model yielding the prior probabilistic density which works as an importance density function. Then, the observation probability density function which is obtained from passes the predicted particles through the non-linear observation model is used to update the importance density particles. Finally, a resampling step is applied to remove the samples with low weights and the posterior probability is redistributed according to the new selected weights. The particles and the corresponding weights prediction and updating are described as follows:
*1*.Initialize with (k = 0)$${\bar{x}}_{0}=x_{0},{w^{i}}_{0}=\frac{1}{N}$$
For i = 1 …N, the filter particles are drawn for ${\bar{\text{x}}}_{0}^{\text{i}}$ from prior P(${\text{x}}_{0})$; where, ${\bar{\text{x}}}_{0}$ and P(${\text{x}}_{0})$ are the initial state vector and variance-covariance matrix.*2*.*Importance Sampling (k = 1: ∞)*
The prior probabilistic motion density is used as an importance density by passing the state vector samples through the nonlinear mechanization equations
$${x^{i}}_{k,k-1}\sim q(x_{t}:x_{0:k-1},y_{1:k-1})$$*3*.*Measurement Updating*
In the measurements updating step, the time updating samples are passing through the non-linear measurements system to create the observation probability density;
$\left(Z^{i}:z_{k,k-1})\sim P(y_{1:k}:x_{1:k-1}^{i}\right)$For i = 1 …N, the importance weight is evaluated as follow;
$$w_{k}^{i}=w_{k-1}^{i}*\frac{p(y_{k}:x_{1:k-1}^{i})p(x_{t}^{i}:x_{k-1}^{i})}{q(x_{k}:x_{0:k-1},y_{1:k-1})}$$
$p(x_{t}^{i}:x_{k-1}^{i})=N(f(x_{k-1},0),Q_{k-1})$Normalize the importance weights;
$w_{k}^{i}=w_{k}^{i}{\left[\sum_{i=1}^{N}w_{k}^{i}\right]}^{-1}$Estimation the mean state vector;
$${\bar{x}}_{k}=\sum_{i=1}^{N}w_{k}^{i}x_{k,k-1}^{i}$$*4*.*Resampling Step*
In this step, the samples with high weights are selected and redistributed. The multinomial distribution resampling technique is applied as pointed out by [32].


### 3.3. Unscented Particle Filter (UPF)

In addition to the computational cost of employing the traditional PF due to the large samples needed to fit the posterior probability distribution, the major drawbacks of using the traditional PF, is the use of the prior probabilistic motion density as an importance density function. The motion importance density may fail to move the weighted particles toward the high-likelihood regions due to the high drift of low-cost inertial sensors, especially during GPS outages. To overcome these limitations, a bank of UKFs (sigma points generating) is used for each particle to generate the importance density functions. The UKF-based importance density is leading to move the particles $x^{i}$ towards the high-likelihood regions by producing new particles ${\bar{x}}^{i}$ with included knowledge about the latest observation. The importance sampling step can be modeled as follows [25]:

For each particle *i* = 1…N, a set of sigma points are defined for *j* = 1…n as follow:
$$x_{k-1}^{i,j}=x_{k-1}^{i}+\sqrt{(n+\lambda ){P^{i}}_{k-1}},w^{i,j}=\frac{1}{2(n+\lambda )}$$
$x_{k-1}^{i,j+n}=x_{k-1}^{i}-\sqrt{(n+\lambda ){P^{i}}_{k-1}},w^{i,j}=\frac{1}{2(n+\lambda )}$Sample propagation for each sigma point (time update)
$$z_{k,k-1}^{i,j}=h(x_{k,k-1}^{i,j}),{\bar{z}}_{k,k-1}^{i,j}=\sum_{j=0}^{2n}w^{i,j}y_{k,k-1}^{i,j}$$
$${\bar{x}}_{k,k-1}^{i,j}=\sum_{j=0}^{2n}w^{i,j}x_{k,k-1}^{i,j}$$
$$P_{zk,k-1}^{i,j}=\sum_{j=0}^{2n}{w^{i,j}}_{i}({\bar{z}}_{k,k-1}^{i,j}-z_{k,k-1}^{i,j}){\left({\bar{z}}_{k,k-1}^{i,j}-z_{k,k-1}^{i,j}\right)}^{T}$$
$p_{k,k-1}^{i,j}=\sum_{j=0}^{2n}w^{i,j}({\bar{x}}_{k,k-1}-x_{k,k-1}^{i}){\left({\bar{x}}_{k,k-1}-x_{k,k-1}^{i}\right)}^{T}$Sample update for each sigma point (measurement update)
$${K^{i}}_{k}=p_{k,k-1}^{i,j}{P_{zk,k-1}^{i,j}}^{T}$$
$${\bar{x}}_{k}^{i}=x_{k,k-1}^{i}+K_{k}(z_{k}-{\bar{z}}_{k,k-1}^{i,j})$$
$$P_{k}^{i}=P_{k,k-1}^{i}-K_{k}P_{zk}^{i,j}{K_{k}}^{T}$$
$${\bar{x}}_{k}^{i}\sim q(x_{t}:x_{0:k-1},z_{1:k-1})$$

## 4. Results and Discussion

**Figure 2:**
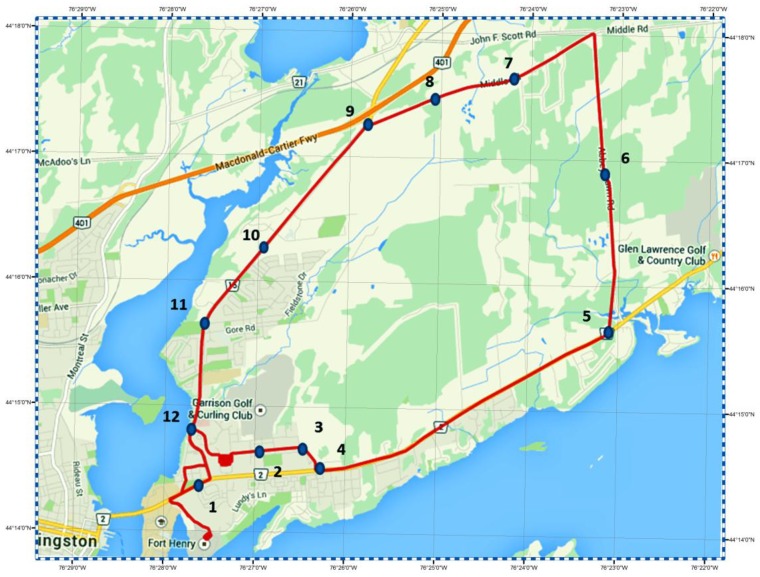
Vehicle test trajectory and simulated complete GPS outages.

A vehicular test was conducted in downtown Kingston, Ontario, to evaluate the performance of the developed integrated GPS-PPP/MEMS-based inertial system. The equipment used comprises the NovAtel SPAN-CPT system and the Trimble R10 GNSS receiver. The SPAN-CPT system consists of NovAtel OEM4 GPS receiver and a MEMS IMU containing three MEMS-based accelerometers and three fiber optic gyros. A differential carrier phase-based GPS/MEMS-based INS solution was obtained to provide the reference solution. In order to create this reference solution, a Trimble R7 GNSS receiver was setup at a point with precisely known coordinates, which was used as a base station. Dual-frequency raw GPS pseudorange, carrier phase and Doppler measurements were logged at a 1 Hz rate, while the IMU raw data were logged at a 100 Hz rate. The duration of the trajectory test was approximately 55 min. Figure 2 shows the trajectory test area.

**Figure 3:**
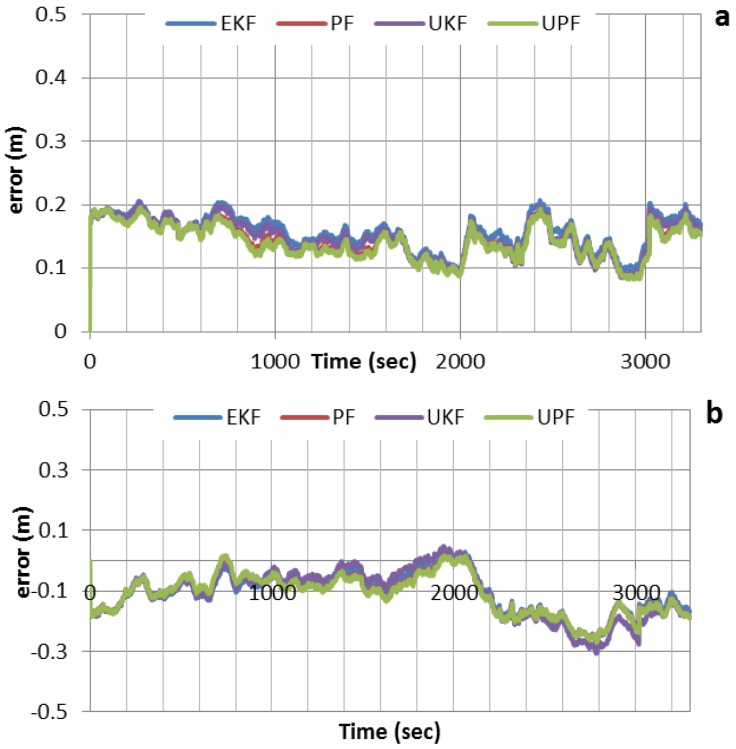

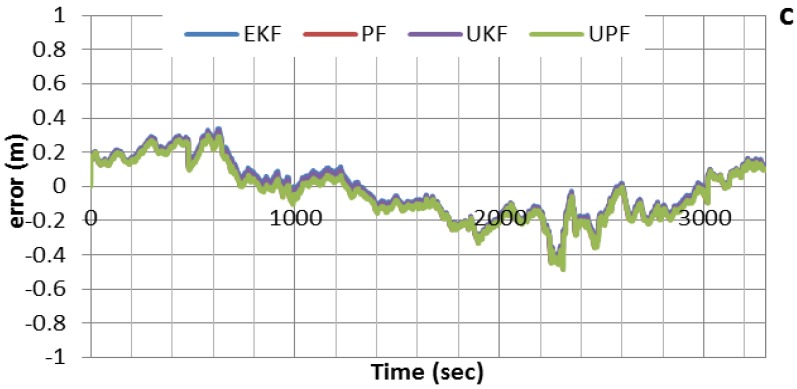
Positioning errors for various filters, with no GPS outages inserted. (**a**) Positioning errors in latitude; (**b**) Positioning errors in longitude; (**c**) Positioning errors in altitude.

Figure 3 shows the positioning solution of the newly developed integrated system for latitude, longitude and altitude, which are compared with the reference solution. As can be seen, all filters can achieve decimeter-level positioning accuracy when no GPS outages are inserted. The results obtained by the various filters agree to the few-centimeter level, which indicate that the effect of non-linearity on the positioning accuracy is marginal. This means that the use of EKF, which is relatively easier to implement, would be advantageous from the estimation cost point of view.

**Figure 4:**
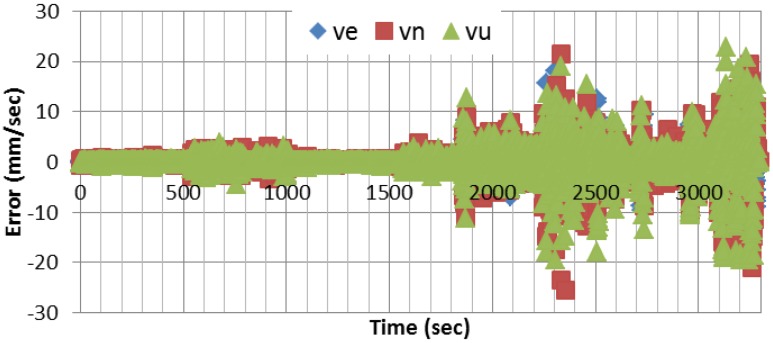
Velocity estimation errors.

**Figure 5:**
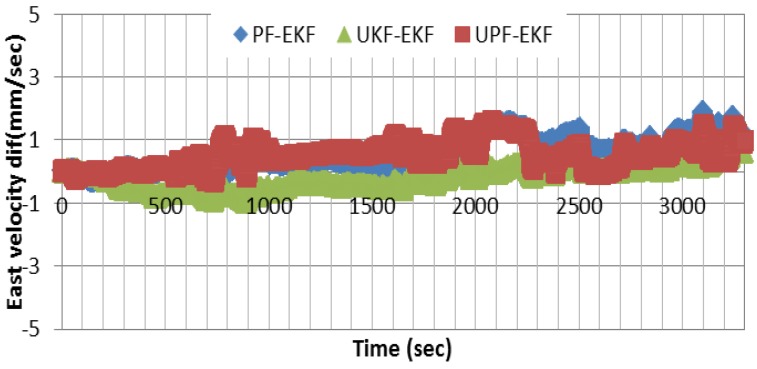
Difference between UKF, PF, and UPF east velocity estimation results and the altitude estimated using EKF.

Figure 4 shows the velocity errors in east, north, and up directions, respectively, using EKF as a central filter. In comparison with the differential mode, the results show that centimeter/sec-level accuracy can be achieved using a single receiver. Figure 5 shows the difference between the east component of the velocity solutions obtained through PF and UKF, respectively, and that of EKF. As can be seen, the solutions agree to the millimeter/sec-level. Similar results are obtained for the other two components.

**Figure 6:**
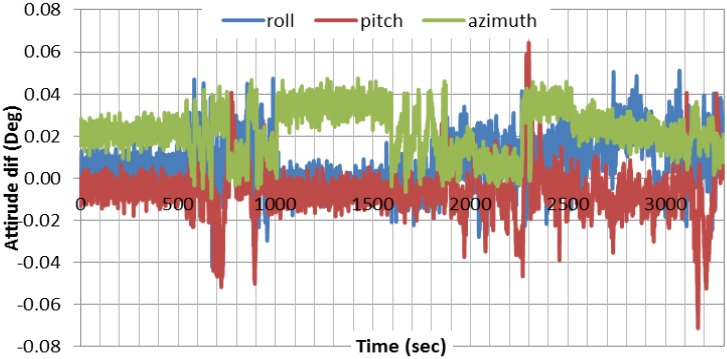
Attitude estimation results using EKF, referenced to differential-based integrated system.

**Figure 7:**
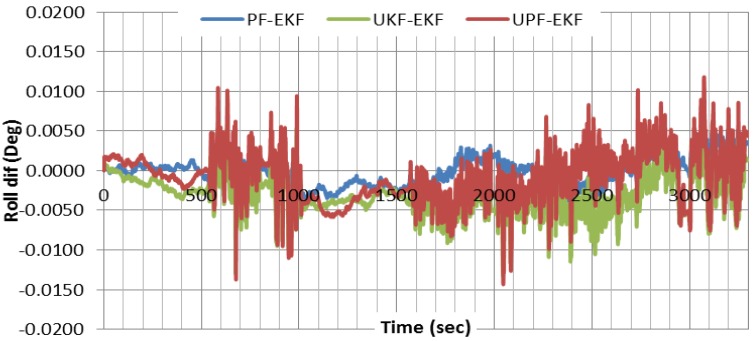
Comparison of roll component results obtained through various estimation filters.

For attitude determination, because of the absence of the external aid in our case, the attitude accuracy depends mainly on the velocity estimation. This is especially correct for the roll and pitch components because of their strong coupling with the horizontal velocities in east and north directions. The accuracy of the estimated azimuth depends mainly on the quality of the gyros used. Figure 6 shows the results of the attitude components, differenced with respect to the differential-based solution, using EKF. As can be seen, the two solutions agree to a high degree of accuracy. Figure 7 compares the roll results obtained through nonlinear filters with those of EKF. As can be seen, all three filters provide comparable roll results. Similar results are obtained for other attitude components.

Based on the positioning, velocity, and attitude results presented above, we can conclude that, when GPS is available, the contribution of the computationally expensive nonlinear filters, such as PF and UPF, is not significant. In other words, EKF, which is relatively easier to implement, would provide a more efficient solution for integrating GPS and MEMS-based inertial measurements.

**Figure 8:**
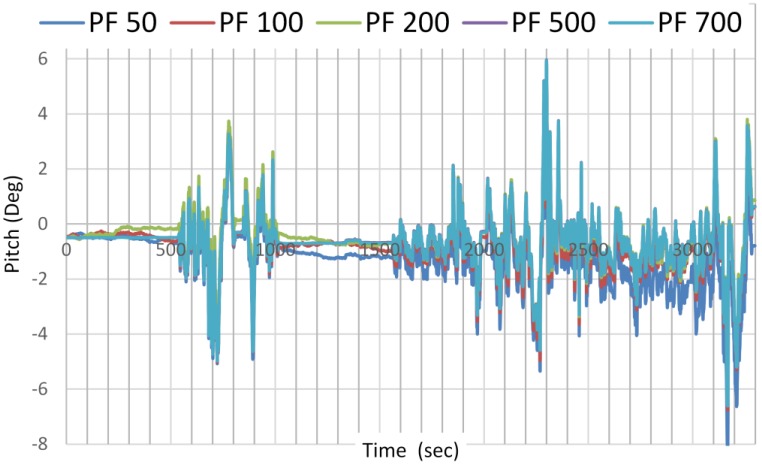
Pitch angle estimation as a function of number of samples used by PF.

**Figure 9:**
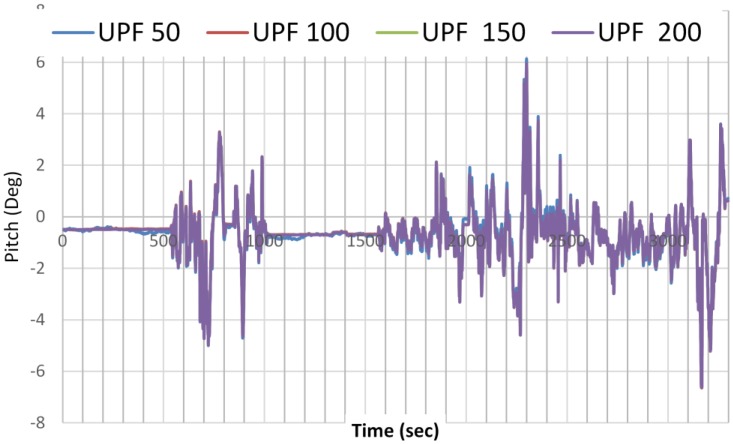
Pitch angle estimation as a function of number of samples used by UPF.

The advantage of using UPF over PF is that the number of particles needed to capture an accurate estimation is reduced. UPF sped up the navigation parameters estimation convergence with small number of particles needed. Figure 8 and Figure 9 show the estimation results of the pitch angle, as an example, on the number of samples needed for PF and UPF. While 500 particles are needed for PF to detect the best estimate of the parameter, only 100 particles are needed for UPF to detect the same. For UPF, increasing the number of particles does not significantly enhance the estimation accuracy.

**Figure 10:**
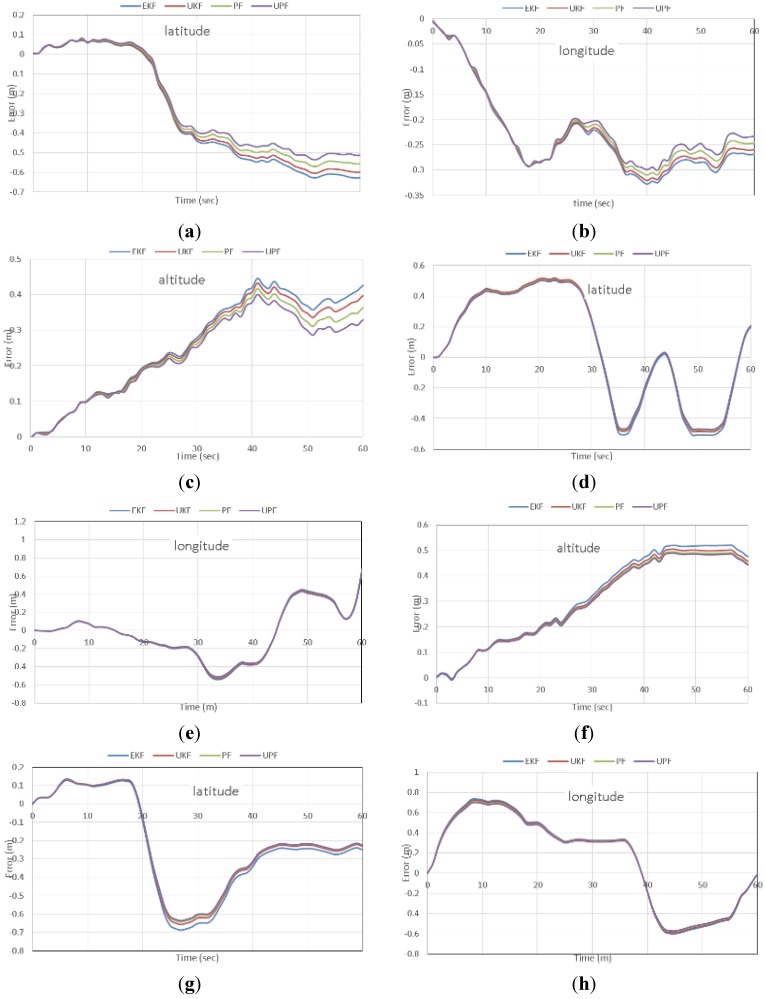

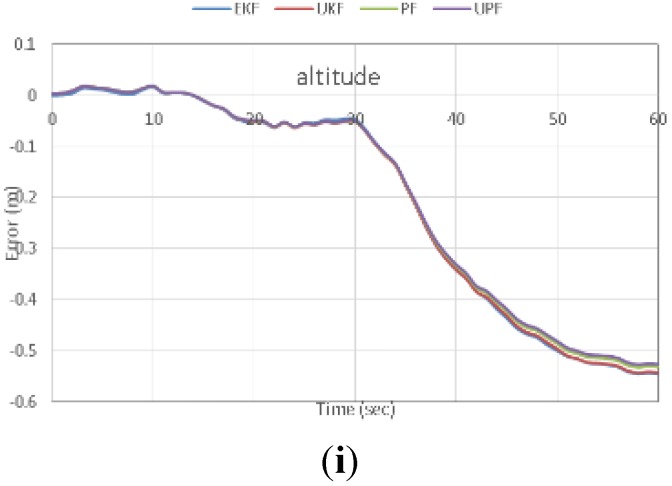
Positioning accuracy during GPS outages for latitude, longitude, and altitude. Outage 2 (**a**–**c**); outage 5 (**d**–**f**) and outage 6 (**g**–**i**).

To simulate challenging positioning conditions through the test trajectory, including high and low speeds, twelve simulated GPS outages of 60 s each are introduced as shown in Figure 1. Figure 10 shows the positioning errors during GPS outages number 2, 5 and 6, as examples. It can be clearly seen that meter-level positioning accuracy can be obtained through all estimation filters.

It can be also seen that UPF slightly enhances the positioning accuracy during the GPS outages, in comparison with the traditional estimation filters. In addition, the nonlinear estimation filters failed to present significant improvement in the positioning accuracy compared with the traditional EKF. This is essentially attributed to the use of linear stochastic models, *i.e.*, first order Gaussian Markov process, for all filters to present a unified comparison between the linear and non-linear estimation filters. In addition, we used fiber optic gyros, as opposed to MEMS-based gyros, which exhibit significantly better behavior.

**Figure 11:**
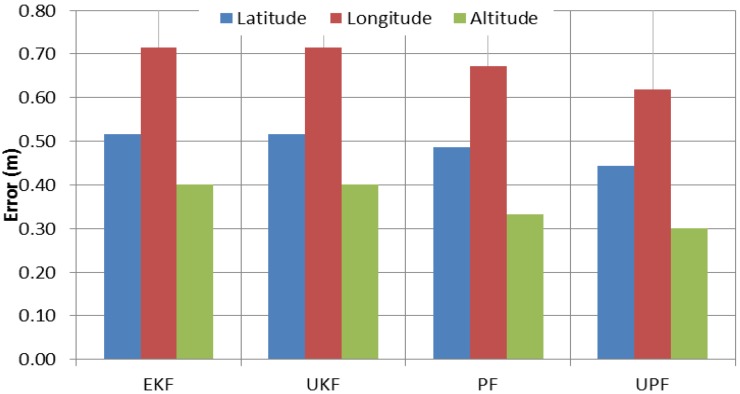
Average of maximum error for different estimation filters during GPS outages.

Figure 11 shows the average of the maximum positioning errors, referenced to the carrier-phase-based DGPS solution, during the 60-second GPS outages. It can be observed that, in comparison with EKF, UPF enhances the positioning accuracy during the GPS outages by 14%, 13% and 15% in latitude, longitude and altitude, respectively. However, compared with PF, the solution improvements are only 6%, 5% and 7% in latitude, longitude and altitude, respectively. It can also be seen that both UKF and EKF present comparable positioning results in all three components.

## 5. Conclusions

This paper examined the performance of UPF and compared its results with those of UKF, the traditional non-linear PF, and the EKF for tightly-coupled PPP GPS/MEMS-based INS integration. A field trial was conducted to evaluate the performance of the developed system. It has been shown that all estimation filters obtain comparable results in positioning, velocity, and attitude, as long as no GPS outages are encountered. However, in comparison with the traditional PF, the use of UPF significantly reduces the number of particles needed to obtain an accurate solution, which speeds up the estimation of navigation parameters. When a complete GPS outage is encountered, the use of UPF enhances the positioning accuracy by up to about 15% in comparison with other estimation filters.
